# Effects of Combined Low-Dose Spironolactone Plus Vitamin E versus Vitamin E Monotherapy on Lipidomic Profile in Non-Alcoholic Fatty Liver Disease: A Post Hoc Analysis of a Randomized Controlled Trial

**DOI:** 10.3390/jcm13133798

**Published:** 2024-06-28

**Authors:** Anastasios Semertzidis, Thomai Mouskeftara, Helen Gika, Petros Pousinis, Kali Makedou, Antonis Goulas, Jannis Kountouras, Stergios A. Polyzos

**Affiliations:** 1 First Laboratory of Pharmacology, School of Medicine, Aristotle University of Thessaloniki, 541 24 Thessaloniki, Greece; agoulas@auth.gr; 2 Laboratory of Forensic Medicine & Toxicology, School of Medicine, Aristotle University of Thessaloniki, 541 24 Thessaloniki, Greece; mousthom@auth.gr (T.M.); gkikae@auth.gr (H.G.); 3 BIOMIC AUTh, Center for Interdisciplinary Research and Innovation, Aristotle University of Thessaloniki, 570 01 Thessaloniki, Greece; petpousinis@hotmail.com; 4 Laboratory of Biochemistry, AHEPA University Hospital, School of Medicine, Aristotle University of Thessaloniki, 541 24 Thessaloniki, Greece; kmakedou@auth.gr; 5 Second Medical Clinic, Ippokration General Hospital, School of Medicine, Aristotle University of Thessaloniki, 546 42 Thessaloniki, Greece; jannis@auth.gr

**Keywords:** lipidomics, nonalcoholic fatty liver disease, randomized controlled trial, spironolactone, treatment, vitamin E

## Abstract

**Background/Objectives**: Lipid dysmetabolism seems to contribute to the development and progression of nonalcoholic fatty liver disease (NAFLD). Our aim was to compare serum lipidomic profile between patients with NAFLD having received monotherapy with vitamin E (400 IU/d) and those having received combination therapy with vitamin E (400 IU/d) and low-dose spironolactone (25 mg/d) for 52 weeks. **Methods**: This was a *post hoc* study of a randomized controlled trial (NCT01147523). Serum lipidomic analysis was performed in vitamin E monotherapy group (*n* = 15) and spironolactone plus vitamin E combination therapy group (*n* = 12). We employed an untargeted liquid chromatography–mass spectrometry lipid profiling approach in positive and negative ionization mode. **Results**: Univariate analysis revealed 36 lipid molecules statistically different between groups in positive mode and seven molecules in negative mode. Multivariate analysis in negative mode identified six lipid molecules that remained robustly different between groups. After adjustment for potential confounders, including gender, omega-3 supplementation, leptin concentration and homeostasis model assessment—insulin resistance (HOMA-IR), four lipid molecules remained significant between groups: FA 20:5, SM 34:2;O2, SM 42:3;O2 and CE 22:6, all being higher in the combination treatment group. **Conclusions**: The combination of spironolactone with vitamin E led to higher circulating levels of four lipid molecules than vitamin E monotherapy, after adjustment for potential confounders. Owing to very limited relevant data, we could not support that these changes in lipid molecules may be beneficial or not for the progression of NAFLD. Thus, mechanistic studies are warranted to clarify the potential clinical significance of these findings.

## 1. Introduction

Nonalcoholic fatty liver disease (NAFLD) is initially characterized by accumulation of fat in the hepatocytes (nonalcoholic fatty liver, NAFL), which may advance to an inflammatory condition, known as nonalcoholic steatohepatitis (NASH) [1]. NASH can progress to liver fibrosis and cirrhosis, which further predispose to the development of hepatocellular carcinoma [2]. Despite its high burden and concerning impact on health globally, there are currently no specifically approved medications for NAFLD, making lifestyle modifications, such as diet and exercise, the cornerstone for its management [3].

Lipid dysmetabolism plays a significant role in the development and progression of NAFLD [4]. NAFLD is characterized by atherogenic dyslipidemia, postprandial lipemia, hypertriglyceridemia and low high-density lipoprotein-cholesterol (HDL-C) concentrations [5]. Thus, NAFLD patients are at high cardiovascular (CV) risk and the first cause of death among them are CV diseases [6].

Lipidomics is a subset of metabolomics that focuses on the comprehensive study and analysis of cellular lipids [7]. Lipidomic studies allow to determine lipid concentrations in tissues and fluids, as well as to discriminate the fatty acid composition of different lipid species, giving valuable information regarding lipid modifications that are involved in the pathophysiology of various diseases, including NAFLD [8]. Even though significant efforts have been made towards the investigation of lipidomic alterations in patients with NAFLD, studies assessing the effect of pharmaceutical interventions on NAFLD lipidomics are scarce.

Vitamin E is a lipid-soluble compound found in various forms, primarily categorized into two main families: tocopherol and tocotrienol [9]. It exerts antioxidant activity through neutralizing free radicals and inhibiting the production of reactive oxygen species, while it is also implicated in signal transduction pathways, gene expression and anti-inflammatory response [10]. According to almost all guidelines, vitamin E can be administered off-label in selected patients with NASH and significant fibrosis [11]. Its long-term use (i.e., over two years) should be avoided due to safety concerns, including increased risk of prostate cancer in men and hemorrhagic stroke; importantly, vitamin E was shown to improve hepatic steatosis and inflammation, but not hepatic fibrosis [12].

Spironolactone is a non-selective mineralocorticoid receptor antagonist, which blockades the renin-angiotensin-aldosterone system (RAAS). In this regard, the stimulation of the RAAS has been suggested to be implicated in the pathogenesis of insulin resistance and NAFLD [13]. Evidence speculating improvement in NASH and hepatic fibrosis through RAAS blockade, along with the favorable impact of spironolactone on myocardial fibrosis, makes spironolactone, an inexpensive medication, a reasonable alternative to be studied in patients with NAFLD [13,14,15]. The effect of vitamin E and/or spironolactone on lipidomic profile in patients with NAFLD or other diseases has not been investigated so far.

The main aim of this *post hoc* study was to compare serum lipidomic profile between biopsy-proven patients with NAFLD having received monotherapy with vitamin E (400 IU/d) vs. those having received combination treatment with vitamin E (400 IU/d) and low-dose spironolactone (25 mg/d) for 52 weeks.

## 2. Materials and Methods

### 2.1. Patients

At the initial randomized controlled trial (RCT; clinicaltrials.gov ID: NCT01147523), 31 patients with biopsy-proven NAFLD were randomized to receive either vitamin E monotherapy (400 IU/d, *n* = 17) or spironolactone (25 mg/d) plus vitamin E (400 IU/d) combination (*n* = 14) treatment for 52 weeks [16]. The preliminary results of this RCT were published in 2011 [17] and its final results in 2017 [16]. For the purpose of the current *post hoc* study, sera of 27 of 31 initially recruited patients, which had been stored at −70 °C, were thawed and untargeted lipidomics analysis was performed. These measurements correspond to the endpoint of the RCT (52 weeks of treatment). No further patient recruitment nor extra intervention was made for the needs of this *post hoc* study. All patients provided informed consent and the study protocols for the initial, as well as for this *post hoc* analysis, were approved by the bioethics committee of the School of Medicine, Aristotle University of Thessaloniki, Greece.

### 2.2. Serum Lipidomic Analysis

For lipidomic analysis, 375 µL of methanol and 1250 µL of methyl tert-butyl ether were added to 50 µL of serum samples, followed by vortexing. Samples were shaken for 30 min at room temperature. Phase separation was enhanced by adding 375 µL of H_2_O and the samples were shaken for 10 min at room temperature. After the incubation period, samples were centrifuged for 10 min at 4 °C and 10,000 rpm. Twelve hundred (1200) μL of the organic phase was transferred to 2 mL Eppendorf tubes and evaporated to dryness in a vacuum (Eppendorf concentrator, Eppendorf SE, Hamburg, Germany). The dried samples were reconstituted with isopropanol (IPA) (1500 μL) for both negative and positive ionization mode. The injection volume was 5 μL for positive ionization and 10 μL for negative ionization. A Quality Control (QC) sample was prepared as a representative by mixing equal volumes of each serum sample. Group-specific QC samples for vitamin E treated group and vitamin E plus spironolactone treated group were also prepared to enhance the identification procedure. Diluted QCs (1:2, 1:4, 1:8) in IPA were also prepared and analyzed to evaluate the dilution integrity of the detected lipids.

### 2.3. Liquid Chromatography–Mass Spectrometry Analysis

Chromatographic analysis was performed with a high-performance liquid chromatography (HPLC) Elute system equipped with an Elute autosampler (Bruker, Billerica, MA, USA). The mobile phase consisted of a binary solvent system, in which solvent A was AcN-H_2_O, 50:50 (*v*/*v*), and solvent B was IPA-AcN-H_2_O, 85:10:5 (*v*/*v*), both containing 5 mM ammonium formate and 0.1% formic acid. The separation of lipids was performed on an Acquity UPLC CSH C18, 2.1 × 100 mm, 1.7 μm column (Waters Ltd., Elstree, UK) at a flow rate of 0.3 mL/min and temperature of 50 °C. A pre-column of the same material was also used.

The mass spectrometry (MS) data were acquired using a TIMS TOF mass spectrometer (Bruker, Billerica, MA, USA), in positive and negative ionization mode. Data-dependent acquisition was performed to enhance the annotation procedure. The parameters were set as follows; source parameters: end plate offset was set to 500 V, while the capillary voltage at ±4500 V. The nitrogen as the dry gas was run with the rate of 10 L/min at dry temperature of 200 °C. The nebulizer gas was set at 2.0 bar. The peak detection threshold was set to 100 counts.

### 2.4. Lipids Identification and Quantification

After acquisition of high-resolution analysis by UHPLC-TOF-MS/MS (Bruker, Billerica, MA, USA), centroided data were recalibrated using sodium formate clusters. Raw data files were converted to analysis base file (ABF) by ABF Converter and imported to MS-DIAL version 4.9 (RIKEN Center for Sustainable Resource Science, Wako, Saitama Prefecture, Japan). The settings for peak picking and feature alignment were as follows: mass accuracy was set to 0.01 Da for MS1 and 0.05 Da for MS2 level data for the purposes of data collection and alignment. Features in the mass range of 50–1500 *m*/*z* and a retention time range from 0–30 min were accepted. Data were smoothed using a linear moving average filter with a box size of 3 and a minimum peak width of 5 datapoints. Protonated species, sodium and ammonium adducts were considered as ion species for peak picking in the positive ion mode; deprotonated species, formic adducts, and water losses were considered as ion species for peak picking in the negative ion mode. Moreover, data were aligned with a retention time tolerance of 0.1 min and a mass tolerance of 0.015 Da. LipidBlast library (Fiehn Lab, UC Davis, Davis, CA, USA) was used for the identification of lipids in both positive and negative ionization mode. Peak areas of all ions in both ionization modes were exported from MS-DIAL after LOESS normalization and were further processed for advanced statistical analysis.

### 2.5. Data Analysis and Visualization

Statistical analyses were conducted in GraphPad Prism version 9.0.0 (GraphPad Software, Boston, MA, USA) and R version 4.3.0 (R Foundation for Statistical Computing, Vienna, Austria). The level of statistical significance was set at *p*-value < 0.05 for all tests. *T*-tests were performed in the univariate analysis to compare lipid molecules between the two groups. Lipid abundances are expressed as normalized peak areas. Lipids with relative standard deviation (RSD)% > 30 in QC samples were filtered out from the statistical analysis. *p*-values were adjusted for multiple comparisons (q-value) using the Benjamini-Hochberg method, with false discovery rate (FDR) setting to <5%. Volcano plots were used to visualize the findings of univariate analysis, in order to provide graphic representations of the differences observed between the two groups.

Further investigation was performed using multivariate analysis techniques, including principal component analysis (PCA) and partial least squares discriminant analysis (PLS-DA) for dimensionality reduction and discovery of influential molecules [18]. PLS-DA models were trained on pareto-scaled and log_10_-transformed peak area data, aiming to distinguish the two groups according to the most informative combinations of lipids (predictive components). The predictive performance of the models was assessed by the Q2Y metric; the higher the Q2Y, the better the performance. Permutation testing was used to estimate the significance of Q2Y (pQ2). Variable importance in projection (VIP) was the metric used for the selection of influential lipids (the higher the VIP score of a particular lipid, the greater its importance) [19]. The threshold of important VIP was set to >1. PCA and PLS-DA are commonly used tools to handle high-dimensional data or the so-called “small n, large *p*” problem, when the number of measured variables (lipids) is much greater than the number of observations (patients). They can provide easily interpretable visualizations and aid in the feature selection process, by identifying variables that contribute the most to the discrimination of the desired class attribute (group). PCA is an exploratory method that uses no prior assumptions, while PLS-DA is a supervised learning method with predefined class labels, which allows better classification between groups. In order to minimize the risk of overfitting observed in PLS-DA, a 7-fold cross-validation technique that divides the dataset into seven folds and repeatedly uses a different fold as the test set was employed, while the remaining folds serve as the training set for the model. Afterwards, the aggregate model metrics were used to estimate the overall performance of the model. Additionally, permutation testing with randomly assigned class labels was performed to ensure reliability of the model.

Finally, linear regression models were built to adjust the obtained results for potential clinical and laboratory confounders. Each lipid molecule that was shown to be significantly different between groups in the univariate or multivariate analysis was handled as the dependent variable in the model. In order to select variables being potential confounders, a selection process was instrumented, also taking into consideration existing knowledge and clinical importance of candidate variables. Univariable linear regression was performed for each lipid against each possible predictor and those with *p*-value < 0.1 were prioritized for multivariable regression. We conventionally used the same potential confounders in the regression models of all lipids to achieve better comparability of the results.

## 3. Results

Lipidomic analyses were conducted in serum samples of 27 patients (week 52), 15 from the vitamin E treated group (9 women) and 12 from the vitamin E plus spironolactone treated group (11 women). The two groups in the primary study were similar in baseline clinical and biochemical characteristics [16]. This was validated for the included patients in this *post hoc* analysis, as it is presented in Table 1. Patient characteristics, including comorbidities and concomitant medications are more extensively described in the original articles of this RCT, published elsewhere [16,17].

In positive and negative ionization modes, 13,077 and 2943 features, respectively, were initially detected. Of them, 129 in positive mode and 85 in negative mode were excluded from the statistical analysis, because of RSD% > 30 in QC samples.

### 3.1. Univariate Analysis

Univariate analysis revealed 36 lipid molecules statistically different between the two groups in positive mode. Most of these lipid molecules belong to the phosphatidylcholine or triglyceride class. In negative mode, seven molecules differed significantly between the two groups. These results are presented in Table 2 and Table 3, respectively, showing the q-value and fold-change for every lipid, as well as the RSD% of each lipid molecule in QC samples. In order to provide a graphic presentation of the differences observed between the two groups, volcano plots were created based on the log_2_ of the fold-change (*x*-axis) and the negative log_10_ of the q-value (*y*-axis) of every identified molecule (Figure S1 depicts the volcano plot for positive mode and Figure S2 for negative mode).

### 3.2. Multivariate Analysis

**Figure 1:**
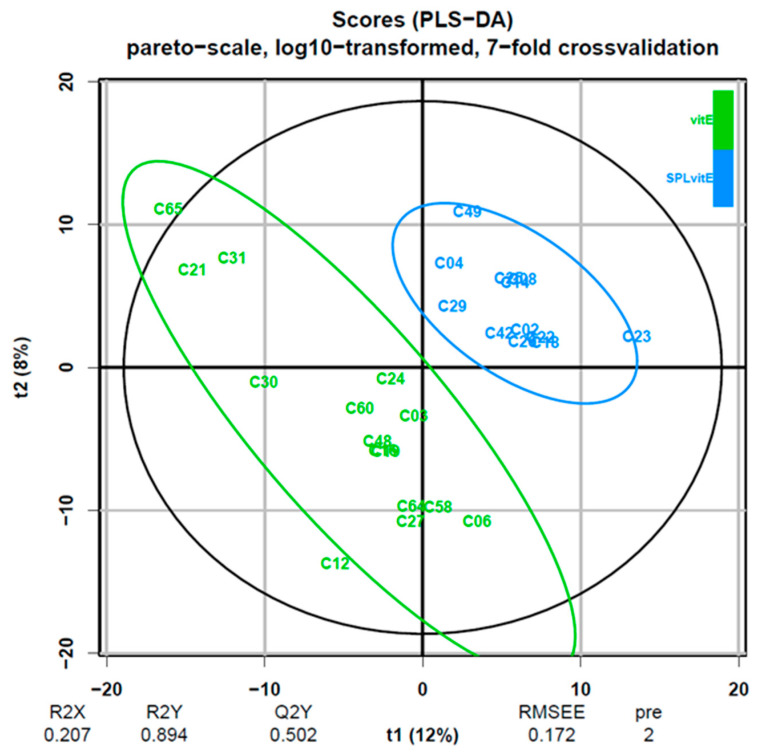
Score plot showing the location of every individual patient (data point) in the reduced dimensional space defined by the first (*x*-axis) and second (*y*-axis) predictive component of the model. Color represents the group in which each patient belongs. Clear separation is observed at the plot, indicating that the model can successfully discriminate the two groups. Model metrics are written at the bottom. Abbreviations: pre, number of predictive components; Q2Y, predictive performance of the model; R2X, proportion of variability in the predictor variables (lipid molecules) that is explained by the model; R2Y, proportion of variability in the response variable (group) that is explained by the model; RMSEE, root mean square error of estimation; SPLvitE, spironolactone plus vitamin E treated group; t1 (%), variance captured by the first predictive component; t2 (%), variance captured by the second predictive component; vitE, vitamin E treated group.

**Figure 2:**
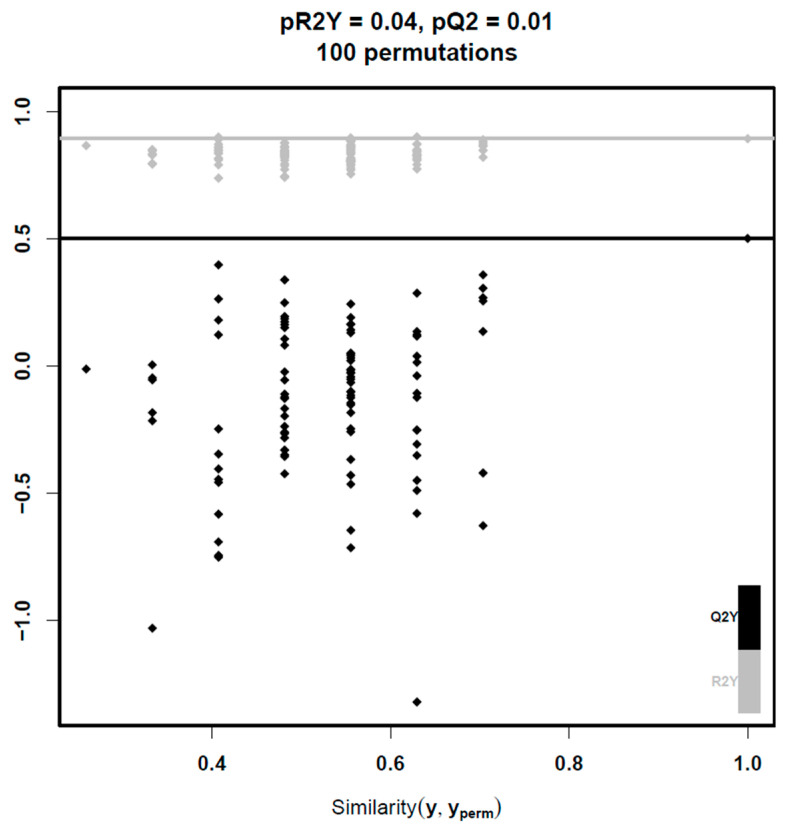
Permutation plot showing diagnostic metrics of models trained after randomly assigning group labels (100 permutations). Horizontal lines correspond to the R2Y and Q2Y of the model trained with the true labels. The proportion of permuted models with a higher Q2Y than the original model (pQ2 = 0.01) confirms the statistical significance of the predictive performance of the model (pQ2 < 0.05). Abbreviations: pQ2, proportion of Q2Y_perm_ above Q2Y; pR2Y, proportion of R2Y_perm_ above R2Y; Q2Y, predictive performance of the model; R2Y, proportion of variability in the response variable that is explained by the model; Similarity (y, y_perm_), similarity between the original and permuted response.

Multivariate analysis encompassed machine learning techniques to deal with the high-dimensional nature of our data. PCA showed significant overlap between the two groups in the feature space and did not manage to efficiently separate them. At the next step, PLS-DA was performed. A valid PLS-DA model with two predictive components (pQ2 = 0.01) was built only for lipids in negative mode leading to potent differentiation between the two groups (Figure 1 and Figure 2). To ensure robustness and reliability of the model, a 7-fold cross-validation technique was implemented for its training and permutation testing to prove that the performance of the model was not due to chance. Using a VIP threshold > 1, the number of lipid molecules that were selected was 775. Afterwards, multiple *t*-tests with FDR correction led to the identification of six lipid molecules that remained robustly different between groups, which are presented in Table 4. In the attempt to train a PLS-DA model on data from positive mode, no significant model was built, because the first predictive component of the PLS-DA model was already not significant.

### 3.3. Linear Regression Models

At the final step of our analysis, for each of the 46 lipid molecules that were significant between groups (36 in univariate analysis of positive mode, 7 in univariate analysis of negative mode and 6 in multivariate analysis of negative mode; 2 molecules in negative mode were significant in both the univariate and multivariate analysis; 1 molecule was significant in both univariate analysis of positive mode and multivariate analysis of negative mode, therefore its negative adduct was selected as its representative), multivariable linear regression analysis was carried out after adjustment for gender, omega-3 supplementation, leptin concentration and HOMA-IR. Group (vitamin E monotherapy vs. combination therapy) was also included, since this was the primary focus of this study. In univariable linear regression analysis, the predefined significance criterion (*p*-value < 0.1, as stated in the methods section) for omega-3 supplementation, gender, leptin concentration and HOMA-IR, was met in 23, 17, 14 and 12 out of the 46 lipid molecules, respectively.

Multivariable models with an overall *p*-value < 0.05 were built for 14 lipid molecules, but the group variable remained significant for only 4 of them, namely FA 20:5 (eicosapentaenoic acid; EPA), SM 34:2;O2, SM 42:3;O2, and CE 22:6 (cholesteryl docosahexaenoate), all being higher in the combination treatment group. Regression coefficients along with their 95% confidence intervals and *p*-values are presented in Tables S1–S4.

## 4. Discussion

This *post hoc* analysis was the first attempt to compare the lipidomic profile of NAFLD patients having received combination therapy of low-dose spironolactone plus vitamin E vs. vitamin E monotherapy for 52 weeks. We employed an untargeted approach, coupled with machine learning methods for data analysis, aiming to uncover the potential effects of spironolactone on circulating lipid molecules and identifying molecules that may possibly have clinical significance.

Only a few studies have assessed the effect of spironolactone or other mineralocorticoid receptor (MR) inhibitors on lipid metabolism, and they were all conducted in populations or conditions different than NAFLD. No effect on circulating lipid levels of healthy adult males was found by Krug et al. after MR antagonism by low-dose eplerenone [20]. A meta-analysis concerning patients with hypertension and diabetes also showed no difference in lipid profile between spironolactone and placebo in patients with arterial hypertension and diabetes [21]. Other authors reported a decrease in serum free fatty acids (FFAs) in women with polycystic ovary syndrome after treatment with spironolactone, but not with metformin or magnesium oxide, in a small 12-week study [22]. In our initial RCT, we found no difference between group* × time interaction in serum lipid profile (i.e., total cholesterol, triglycerides, HDL-C, LDL-C) [16]. However, all previous studies have investigated the effect of MR inhibitors on clinically relevant lipid profiles, and not on multiple lipid molecules as we did in this study.

In this *post hoc* analysis study, only a small proportion of the whole lipidome differed significantly between the two groups, mainly consisting of phosphatidylcholines and triglycerides, followed by sphingomyelins, fatty acids, and cholesteryl esters. This proportion was further reduced after adjustment for potential confounders, being gender, omega-3 supplementation, leptin concentration, and HOMA-IR. Omega-3 supplementation may directly affect the levels of some lipids [23,24]. Leptin might influence lipidomic profile, as it has been shown to modulate lipid metabolism and distribution in various tissues including the liver, skeletal muscle, and adipose tissue [25]. Insulin resistance leads to dysregulation of lipolysis in adipocytes and increased supply of FFAs to the liver, which may modify the measured levels of fatty acids in serum [17]; HOMA-IR was used as a marker of insulin resistance. At the end of robust analysis presented above, FA 20:5, SM 34:2;O2, SM 42:3;O2, and CE 22:6 were significantly higher in the group of the combination treatment of spironolactone and vitamin E than the group of monotherapy with vitamin E, after adjustment for the aforementioned potential confounders.

FA 20:5 or EPA, is an omega-3 (n-3) fatty acid; EPA was shown to decrease along with other long-chain polyunsaturated fatty acids (PUFAs) in the liver and the circulation of patients with NASH [26,27], but this is not a constant finding, since other authors showed similar EPA levels between patients with NAFL and NASH [1]. Impaired activity of fatty acid desaturase 1 and elongation of very long chain fatty acids 6, which are key enzymes in fatty acid synthesis, have been demonstrated in the liver of NASH patients, explaining the depletion of n-3 PUFAs and increased n-6/n-3 ratio that has also been reported in other lipidomic studies [4,28]. It has been suggested that elevated n-6/n-3 PUFA ratio leads to aggravation of hepatic inflammation, as more eicosanoids with proinflammatory properties are synthesized at the cost of pro-resolving mediators that normally alleviate ER stress, oxidative stress, and apoptosis [7,29]. In this regard, the enhancement of n-3 fatty acid levels in the liver was shown to lead to improvement in steatosis and insulin resistance, as well as in lipid peroxidation and necroinflammation in a mouse model of NASH [30]. Additionally, EPA concentrations along with other six eicosanoids have been associated with fibrosis improvement in a clinical trial of selonsertib and simtuzumab in NASH patients [31]. Even though restoration of n-6/n-3 ratio is a plausible therapeutic approach, evidence from clinical trials does not suggest omega-3 PUFA supplementation as a therapeutic approach for NASH patients [3]. Despite this failure of omega-3 PUFA supplementation, the observed increase in EPA levels after treatment with the combined regimen in our study may indicate a beneficial effect of spironolactone towards this direction, which, however, should be validated in liver tissue and further investigated in mechanistic studies.

Sphingolipid metabolism alterations have also been associated with alterations in the pathogenesis of NAFLD [32]. The main contributors of this lipid class to the disease are ceramides, which are known to promote insulin resistance through inhibition of protein kinase B-mediated insulin signaling; ceramides have also been associated with toll-like receptor 4 dependent inflammation [7,33]. Ceramides can be synthesized de novo or by the enzyme sphingomyelinase through hydrolysis of sphingomyelin (SM), which is the most abundant sphingolipid in the plasma membrane and lipoproteins [32,33]. Therefore, ceramide concentrations are affected even by small changes in SM abundancy [33]. Findings from lipidomic studies regarding SM concentrations and their association with NAFLD severity are contradictory. Tiwari-Heckler et al. reported an overall increase in serum SM content of NAFL and NASH patients compared to healthy controls [34], while Zhou et al. identified a cluster of circulating SMs that were significantly reduced in NASH patients compared to non-NASH individuals [35]. A previous lipidomic case-control study of our group found lower concentration of SMs in NASH, but not NAFL, patients compared to non-NAFLD controls [1]. In an older study of individuals with obesity by Barr et al., seven SM species (SM 36:3, d18:2/16:0, d18:2/14:0, d18:1/18:0, d18:1/16:0, d18:1/12:0, d18:0/16:0) showed significant alterations between NAFLD patients and non-NAFLD controls [36]. Interestingly, SM d18:2/16:0 (34:2) was decreased in NAFLD group in this study. In this regard, the increase in SM 34:2;O2 concentration, as well as that in SM 42:3;O2, in the combination treatment group, we observed in this study, may be further explored in mechanistic studies to clarify whether, first, these changes are reflected in the liver tissue and, second, are advantageous or not in NAFLD patients.

Accumulation of free cholesterol in the hepatocytes during the progression from NAFL to NASH has also been supported [4]. However, there is conflict on the exact role of cholesteryl esters (CE) in NAFLD. Peng et al. reported higher total hepatic CE content in patients with NAFL and NASH, along with an increase in the percentage of docosahexaenoic acid (DHA, FA 22:6) within CE in NASH compared with the controls [37]. On the contrary, Puri et al. detected no difference in total liver CE, but they supported an increase in PUFA content within CE in both NAFL and NASH patients compared with the control group, as well as a non-significant trend of increase in CE 22:6 [26]. Previous results from serum and plasma lipidomic studies are largely in discrepancy with those from liver biopsies, as CE concentration has been shown to decrease in NAFL and NASH patients compared with controls [1]. Gorden et al. discovered a panel of lipid species in plasma distinguishing NASH from NAFL [38]. CE 22:6 was one of the 20 highest-ranked molecules in terms of statistical significance and was found to be reduced in NASH compared with NAFL in this study [38]. Since CE 22:6 was increased in the combination treatment group compared with vitamin E monotherapy in our study, these results warrant further mechanistic studies to clarify whether the potentially increasing effect of spironolactone on circulating CE 22:6 is reflected in the liver tissue and whether this may be accompanied by changes in liver histology. Of course, the validation of our findings in similar studies of spironolactone or other mineralocorticoid receptor antagonists in NAFLD patients or even in NAFLD-related transcriptome databases would have been of importance.

Strengths of our study are its originality, the state-of-the-art methods of lipidomic analysis, and the focus on an older and inexpensive medication (spironolactone), with which probably no pharmaceutical company is interested in setting clinical trials. However, this study has certain limitations. First, the sample size was small, although sufficient to produce statistically significant results after robust statistical analysis; in this regard, the sample was sufficiently powered at least for the variables that were shown statistically significant. Second, this study was a *post hoc* analysis of a previous RCT that was designed towards another primary aim; in this regard, systematic bias might have occurred, which may probably have been avoided, if the RCT was primarily designed towards the aim of this study. Third, this was a non-blinded and open-label RCT, conditions that might have subconsciously affected its results. Furthermore, liver biopsy was not performed at the end of the study, mainly due to unwillingness of most participants to consent to a paired liver biopsy. Additionally, the results of this study cannot be widely generalized until similar trials are performed in patients with NAFLD of other races and age groups. Moreover, the observed changes of lipid species could not be associated with potential improvement in clinical characteristics of the patients; however, almost all patients included in this RCT were asymptomatic at baseline and remained asymptomatic after a 1-year treatment.

In conclusion, this *post hoc* analysis of an RCT showed that the combination of spironolactone with vitamin E, as compared with vitamin E monotherapy, led to higher circulating levels of four lipid molecules, after adjustment for potential confounders, including omega-3 supplementation. Since there are limited data on these specific lipid molecules, mostly from case-control studies, we could not support that the changes in these lipid molecules induced probably by spironolactone may be beneficial for NAFLD in terms of the progression of liver disease, but also in terms of cardiovascular disease, commonly encountered in NAFLD. Thus, mechanistic studies are warranted to clarify the potentially clinical significance of these findings.

## Figures and Tables

**Table 1:** Clinical and biochemical parameters of the patients.

Characteristic	VitE Group (*n* = 15)	SPL + VitE Group (*n* = 12)	*p*-Value †
Women/Men (n)	9/6	11/1	0.09
Omega-3 Supplementation [n (%)]	3 (20%)	2 (17%)	0.99
Age (years)	54.0 ± 3.7	56.8 ± 1.8	0.62
BMI (kg/m^2^)	34.1 ± 1.8	32.3 ± 1.5	0.49
Waist circumference (cm)	110 ± 4	105 ± 3	0.43
Systolic blood pressure (mmHg)	130 ± 5	132 ± 2	0.90
Diastolic blood pressure (mmHg)	83 ± 4	84 ± 2	0.71
AST (U/L)	39 ± 8	29 ± 2	0.94
ALT (U/L)	52 ± 11	36 ± 4	0.51
GGT (U/L)	61 ± 16	37 ± 6	0.94
Total cholesterol (mg/dL)	219 ± 9	231 ± 14	0.57
Triglycerides (mg/dL)	219 ± 31	205 ± 21	0.99
HDL-C (mg/dL)	49 ± 2	51 ± 3	0.61
LDL-C (mg/dL)	127 ± 10	139 ± 11	0.52
Glucose (mg/dL)	106 ± 7	103 ± 6	0.85
Insulin (μU/mL)	12.0 ± 2.5	12.7 ± 3.0	0.83
HOMA-IR	3.2 ± 0.7	3.3 ± 0.7	0.83

Data are presented as mean ± standard error of the mean or numbers and frequencies. † Mann-Whitney U test for continuous variables; Fisher’s exact test for categorical variables. Abbreviations: ALT, alanine aminotransferase; AST, aspartate aminotransferase; BMI, body-mass index; GGT, gamma-glutamyl transferase; HDL-C, high-density lipoprotein-cholesterol; HOMA-IR, homeostasis model assessment insulin resistance; LDL-C, low-density lipoprotein-cholesterol; SPL, spironolactone; VitE, vitamin E.

**Table 2:** Lipid molecules in positive mode being different between groups (univariate analysis).

Lipid Molecule †	Q-Value	FC ‡	RSD% in QC Samples
CE 16:1	<0.001	1.094	3.589
CE 20:4	<0.001	0.821	10.924
CE 20:5	<0.001	1.742	2.988
CE 22:6	<0.001	1.405	6.470
DG 34:1	<0.001	1.106	4.861
LPC 22:6	<0.001	1.309	6.009
PC 32:2	0.042	1.040	5.801
PC 36:5	<0.001	1.072	5.044
PC 38:2	<0.001	1.103	1.572
PC 38:4	0.016	0.955	4.536
PC 38:5|PC 16:0_22:5	<0.001	1.075	3.868
PC 38:5|PC 18:0_20:5	<0.001	1.278	1.910
PC 38:6|PC 18:1_20:5	<0.001	1.380	8.073
PC 38:6|PC 18:2_20:4	0.001	0.915	9.372
PC 40:5	<0.001	1.186	2.177
PC 40:6	<0.001	1.114	5.770
PC O-34:2	<0.001	0.787	0.909
PC O-34:3	<0.001	0.929	1.280
PC O-36:2	0.037	0.833	5.387
PC O-36:5	<0.001	0.903	3.099
PC O-38:4	<0.001	0.911	6.656
SM 40:2;O2	<0.001	1.121	0.851
SM 41:2;O2	<0.001	1.168	6.084
TG 42:1	0.011	1.400	10.674
TG 44:1	<0.001	1.132	11.708
TG 46:1	<0.001	1.149	2.731
TG 46:2	<0.001	1.151	11.023
TG 48:1	0.001	1.040	6.055
TG 48:2	<0.001	1.067	6.009
TG 48:3	0.014	1.065	10.302
TG 50:0	<0.001	0.906	1.938
TG 52:0	0.005	0.828	14.065
TG 52:1	<0.001	1.072	6.095
TG 52:5	<0.001	1.079	13.191
TG 54:6	0.008	0.807	14.337
TG 56:7	<0.001	1.464	11.718

†: Lipid molecules are classified according to their bulk number in alphabetical order. For molecules with the same bulk number, fatty acyl chain composition is indicated on the right of the pipe. ‡: FC > 1 denotes higher concentration in the SPL + VitE group than VitE group, whereas FC < 1 denotes the opposite. Abbreviations: CE, Cholesteryl Ester; DG, Diacylglycerol; FC, fold-change between the two groups (SPL + VitE vs. VitE); LPC, Lysophosphatidylcholine; PC, Phosphatidylcholine; QC, Quality Control; RSD, Relative Standard Deviation; SM, Sphingomyelin; SPL, Spironolactone; TG, Triacylglycerol; VitE, Vitamin E.

**Table 3:** Lipid molecules in negative mode being different between groups (univariate analysis).

Lipid Molecule †	Q-Value	FC ‡	RSD% in QC Samples
FA 20:5	0.024	1.731	3.703
FA 22:6	<0.001	1.364	2.322
LPC 16:0	<0.001	0.989	2.111
LPC 18:0	<0.001	1.017	1.837
PC 34:1	<0.001	1.085	3.446
SM 32:1;O2	<0.001	1.172	3.561
SM 34:2;O2	<0.001	1.141	3.137

†: Lipid molecules are classified according to their bulk number in alphabetical order. ‡: FC > 1 denotes higher concentration in the SPL + VitE group than VitE group, whereas FC < 1 denotes the opposite. Abbreviations: FA, Fatty Acid; FC, fold-change between the two groups (SPL + VitE vs. VitE); LPC, Lysophosphatidylcholine; PC, Phosphatidylcholine; QC, Quality Control; RSD, Relative Standard Deviation; SM, Sphingomyelin; SPL, spironolactone; VitE, Vitamin E.

**Table 4:** Statistically significant lipid molecules in negative mode (multivariate analysis).

Lipid Molecule †	Q-Value	VIP	FC ‡	RSD% in QC Samples
FA 16:1	0.006	1.499	0.886	3.929
FA 20:5	<0.001	1.836	1.731	3.703
FA 22:5	0.046	1.354	1.182	2.635
FA 22:6	<0.001	1.739	1.364	2.322
PC 36:5	<0.001	1.323	1.454	1.390
SM 42:3;O2	<0.001	1.427	1.256	3.679

†: Lipid molecules are classified according to their bulk number in alphabetical order. ‡: FC > 1 denotes higher concentration in the SPL + VitE group than VitE group, whereas FC < 1 denotes the opposite. Abbreviations: FA, Fatty Acid; FC, fold-change between the two groups (SPL + VitE vs. VitE); PC, Phosphatidylcholine; QC, Quality Control; RSD, Relative Standard Deviation; SM, Sphingomyelin; SPL, spironolactone; VIP, Variable Importance in Projection; VitE, Vitamin E.

## Data Availability

The dataset is available by the corresponding authors upon reasonable request, after the permission of their institutions.

## Supplementary Materials

The following supporting information can be downloaded at: https://www.mdpi.com/article/10.3390/jcm13133798/s1, Figure S1: Volcano plot of univariate analysis containing identified lipid molecules in positive mode; Figure S2: Volcano plot of univariate analysis containing identified lipid molecules in negative mode; Table S1: Model summary for FA 20:5; Table S2: Model summary for SM 34:2;O2; Table S3: Model summary for SM 42:3;O2; Table S4: Model summary for CE 22:6.
