# Supplementary material for: Effects of Combined Low-Dose Spironolactone Plus Vitamin E versus Vitamin E Monotherapy on Lipidomic Profile in Non-Alcoholic Fatty Liver Disease: A Post Hoc Analysis of a Randomized Controlled Trial
Source: J Clin Med. 2024 Jun 28;13(13):3798. doi: 10.3390/jcm13133798 (PMC11242225; doi:10.3390/jcm13133798)
Supplement: Supplementary file 1 [file jcm-13-03798-s001.zip › jcm-3033286-supplementary.pdf]

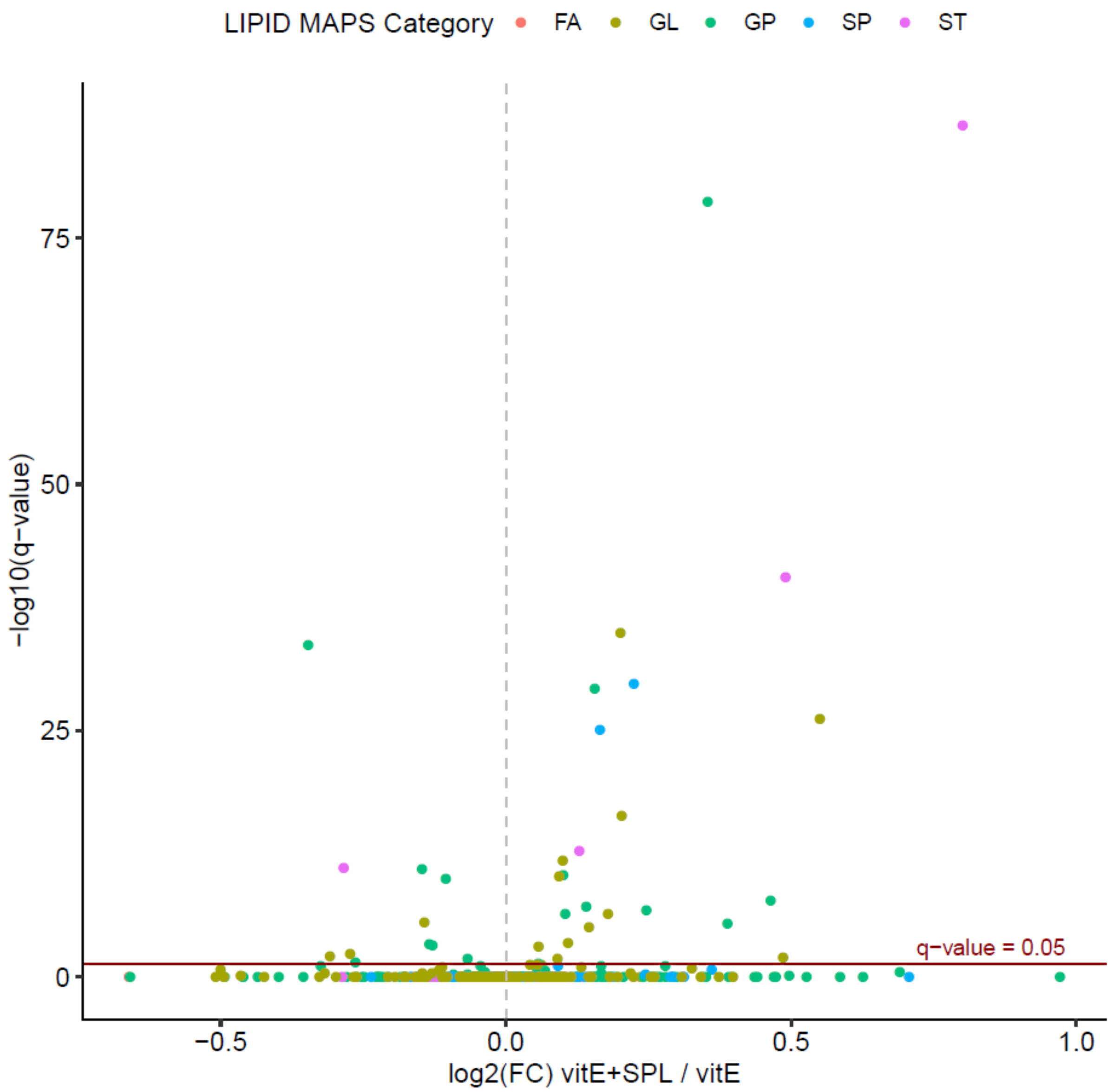

Figure S1. Volcano plot of univariate analysis containing identified lipid molecules in positive mode. Each point represents data of a different lipid molecule. X-axis shows the $\log_2$(fold-change) indicating how much the concentration of each molecule differs between the two groups. Molecules that are located on the right of the vertical, grey, dashed line are higher in the combination treatment group, while those on the left of the line are higher in the vitamin E monotherapy group. Y-axis shows the negative $\log_{10}$(q-value) reflecting the statistical significance of the difference in the concentration of each molecule between the two groups. Molecules located above the horizontal, red line are significantly different between groups. Molecules that exhibit large fold-changes and high statistical significance are located at the upper corners of the plot. Color corresponds to the LIPID MAPS category of each metabolite. Abbreviations: FA, Fatty Acyls; FC, fold-change; GL, Glycerolipids; GP, Glycerophospholipids; SP, Sphingolipids; SLP, spironolactone; ST, Sterol Lipids; VitE, vitamin E.

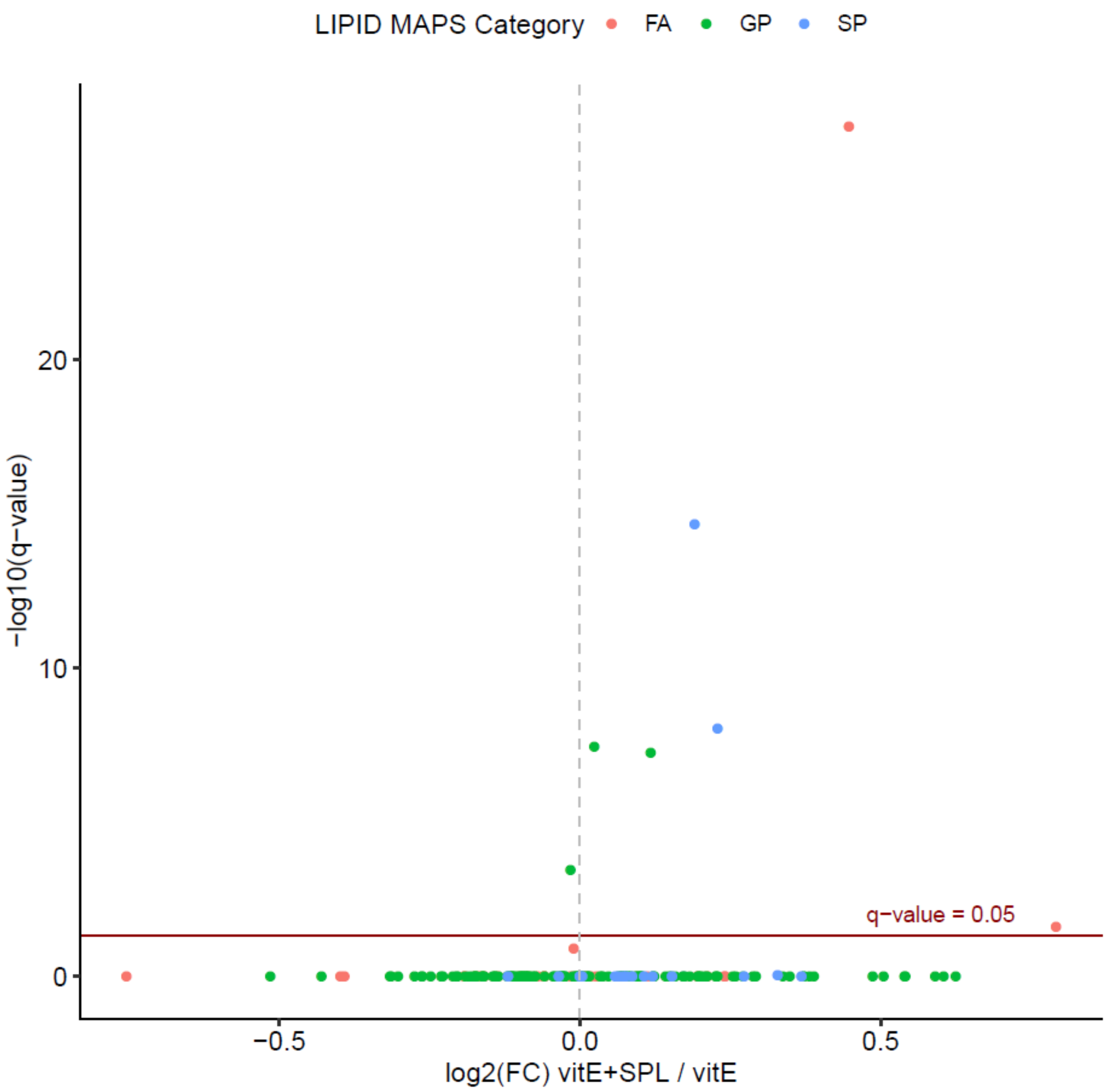

Figure S2. Volcano plot of univariate analysis containing identified lipid molecules in negative mode. Each point represents data of a different lipid molecule. X-axis shows the $\log_2$(fold-change) indicating how much the concentration of each molecule differs between the two groups. Molecules that are located on the right of the vertical, grey, dashed line are higher in the combination treatment group, while those on the left of the line are higher in the vitamin E monotherapy group. Y-axis shows the negative $\log_{10}$(q-value) reflecting the statistical significance of the difference in the concentration of each molecule between the two groups. Molecules located above the horizontal, red line are significantly different between groups. Molecules that exhibit large fold-changes and high statistical significance are located at the upper corners of the plot. Color corresponds to the LIPID MAPS category of each metabolite.

Abbreviations: FA, Fatty Acyls; FC, fold-change; GP, Glycerophospholipids; SP, Sphingolipids; SLP, spironolactone; VitE, vitamin E.

**Table S1. Model summary for FA 20:5**			
**Dependent variable**	**Regression Coefficient**	**95% CI**	**P-value**
Group			
VitE	Reference		
SPL + VitE	0.00042	0.00008, 0.00076	0.018
Omega-3 supplementation			
No	Reference		
Yes	0.00095	0.00052, 0.00138	<0.001
Gender			
Female	Reference		
Male	-0.00018	-0.00065, 0.00030	0.4
Leptin (ng/ml)	0.00001	0.00000, 0.00002	0.2
HOMA-IR	-0.00001	-0.00009, 0.00007	0.9

Abbreviations: CI, Confidence Interval; FA, Fatty Acid; HOMA-IR, Homeostasis Model Assessment for Insulin Resistance; SPL, Spironolactone; VitE, Vitamin E

**Table S2. Model summary for SM 34:2;O2**			
**Dependent variable**	**Regression Coefficient**	**95% CI**	**P-value**
Group			
VitE	Reference		
SPL + VitE	0.00102	0.00018, 0.00186	0.019
Omega-3 supplementation			
No	Reference		
Yes	-0.00022	-0.00128, 0.00083	0.7
Gender			
Female	Reference		
Male	0.00016	-0.00099, 0.00132	0.8
Leptin (ng/ml)	0.00009	0.00006, 0.00012	<0.001
HOMA-IR	-0.00015	-0.00034, 0.00005	0.13

Abbreviations: CI, Confidence Interval; HOMA-IR, Homeostasis Model Assessment for Insulin Resistance; SM, Sphingomyelin; SPL, Spironolactone; VitE, Vitamin E

Table S3. Model summary for SM 42:3;O2			
**Dependent variable**	**Regression Coefficient**	**95% CI**	**P-value**
Group			
VitE	Reference		
SPL + VitE	0.00029	0.00004, 0.00053	0.024
Omega-3 supplementation			
No	Reference		
Yes	-0.00020	-0.00051, 0.00011	0.2
Gender			
Female	Reference		
Male	-0.00006	-0.00040, 0.00028	0.7
Leptin (ng/ml)	0.00001	0.00001, 0.00002	0.003
HOMA-IR	-0.00006	-0.00011, 0.00000	0.052

Abbreviations: CI, Confidence Interval; HOMA-IR, Homeostasis Model Assessment for Insulin Resistance; SM, Sphingomyelin; SPL, Spironolactone; VitE, Vitamin E

**Table S4. Model summary for CE 22:6**			
**Dependent variable**	**Regression Coefficient**	**95% CI**	**P-value**
Group			
VitE	Reference		
SPL + VitE	0.00030	0.00005, 0.00056	0.023
Omega-3 supplementation			
No	Reference		
Yes	0.00042	0.00009, 0.00074	0.014
Gender			
Female	Reference		
Male	-0.00006	-0.00042, 0.00029	0.7
Leptin (ng/ml)	0.00001	0.00000, 0.00002	0.12
HOMA-IR	-0.00004	-0.00010, 0.00002	0.2

Abbreviations: CE, Cholesteryl Ester; CI, Confidence Interval; HOMA-IR, Homeostasis Model Assessment for Insulin Resistance; SPL, Spironolactone; VitE, Vitamin E
